# Evaluation of Vitamin D Levels and Response to Therapy of Childhood Migraine

**DOI:** 10.3390/medicina55070321

**Published:** 2019-06-28

**Authors:** Betül Kılıç, Mustafa Kılıç

**Affiliations:** 1 Department of Child Neurology, University of Health Sciences, Derince Training and Research Hospital, 41900 Kocaeli, Turkey; 2 Department of Neurosurgery, University of Health Sciences, Şişli Hamidiye Etfal Training and Research Hospital, 34371 Istanbul, Turkey

**Keywords:** childhood migraine, vitamin D, pedMIDAS

## Abstract

*Background and Objectives:* Vitamin D deficiency and insufficiency are related with many neurological diseases such as migraine. The aim of this study was to investigate whether pediatric migraine is associated with vitamin D deficiency and the effect of vitamin D therapy on the frequency, duration, severity of migraine attacks, and Pediatric Migraine Disability Assessment (PedMIDAS). *Materials and Methods:* We retrospectively examined the patients’ levels of calcium, phosphorus, parathyroid hormone, alkaline phosphatase, and 25-OH vitamin D of 92 pediatric migraine patients. The patients were divided into two groups: Group 1, which had low vitamin D levels and received vitamin D therapy, and group 2, which had normal vitamin D levels and did not receive vitamin D therapy. Migraine severity measured by the visual analog scale (VAS), migraine frequency, and duration as well as scores on the PedMIDAS questionnaire were compared with regard to the 25-OH vitamin D levels. In addition, pre- and posttreatment pedMIDAS scores, VAS, migraine frequency, and duration were compared with baseline values. *Results:* A total of 34.7% patients had vitamin D insufficiency (vitamin D levels between 10 and 20 ng/mL), whereas 10.8% had vitamin D deficiency (vitamin D levels < 10 ng/mL). Migraine frequency, migraine duration, and PedMIDAS scores were significantly higher in the group 1 than group 2 (*p* = 0.004, *p* = 0.008, and *p* = 0.001). After vitamin D therapy at sixth months of supplementation, migraine duration was reported statistically significant shorter (*p* < 0.001) and the migraine frequency, VAS scores, and pedMIDAS scores were statistically significant lower compared with baseline values in group 1 (*p* < 0.001). *Conclusion:* We found a marked correlation between pediatric migraine and vitamin D levels. Vitamin D therapy was beneficial in migraine pediatric patients.

## 1. Introduction

Migraine is a neurologic condition characterized by hypersensitivity to auditory, olfactory, visual, and cutaneous stimuli; vomiting and nausea; and severe headache. The pathophysiology of migraine is not completely understood; however, the commonly reported hypotheses include central sensitization, neurogenic inflammation, atypical pain processing, and cortical hyperexcitability [1].

As a prohormone, vitamin D plays a critical role in controlling phosphorus and calcium metabolism. In adolescence and childhood, bone health is affected by vitamin D [2]. Ultraviolet B light helps absorb approximately 90% of the vitamin D into the epidermis; the vitamin D is then transformed to 25-OH vitamin D in the liver and 1,25-OH vitamin D in the kidneys [3]. Vitamin D is processed through genomic and nongenomic pathways [4]. The process begins with the binding of vitamin D to the vitamin D receptor (VDR) in the genomic pathway. VDR is a steroid/thyroid member belonging to the superfamily of nuclear transcription factors [5]. According to findings reported based on animal studies, VDR is present in certain brain regions, such as the hippocampus, hypothalamus, amygdala, cortex, thalamus, and cerebellum [6,7].

Recent evidence indicates that various brain disorders, such as epilepsy, Alzheimer’s disease, multiple sclerosis, depression, autism, and schizophrenia, and some immune-mediated disorders, such as type I diabetes mellitus, rheumatoid arthritis, inflammatory bowel diseases, and systemic lupus erythematosus, may arise due to vitamin D imbalance [8].

A series of painful conditions are linked to vitamin D deficiency [9]. Few recent studies have demonstrated that tension-type headache and migraine may be related to low vitamin D serum levels [3,8,10]. According to few small-scale studies and reports, vitamin D therapy is beneficial for headache disorders [11,12,13,14,15]. However, very few pediatric reports are available [16,17,18].

In this study, we aimed to clarify whether migraine and vitamin D deficiency are related in pediatric patients and to understand the effect of vitamin D therapy in providing relief from migraine.

## 2. Materials and Methods

This retrospective study included patients with episodic migraine who were admitted to the pediatric neurology outpatient clinic of Kocaeli Derince Training and Research Hospital between May 2017 and September 2018. Migraine (without aura) was diagnosed according to the criteria established in the International Classification of Headache Disorders, third edition, beta version (ICHD-3 beta) of the International Headache Association [19].

The exclusion criteria were as follows: patients with signs and symptoms of chronic diseases (e.g., liver, skin, metabolic, renal, gastrointestinal, or bone disease; psychiatric disorder; cerebral palsy; and cognitive/intellectual disability), those with diseases causing immobilization and malabsorption, those with dietary restrictions, those with osteoporosis, those receiving any medication affecting bone metabolism (vitamin D, calcium, steroids, or nonsteroidal anti-inflammatory drugs), those with incomplete laboratory results, and those who had taken any prophylactic or other regular medication.

Demographic data was collected for the study including family history, sex, and age. The patients were evaluated with regard to their migraine duration and frequency. The duration of the migraine headache was determined as hours in the day. The migraine frequency was determined as the number of migraine attacks within one month. Serum levels of 25-OH vitamin D, calcium (Ca), parathormone (PTH), alkaline phosphatase (ALP), and phosphorus (P) results were reviewed. The Migraine Disability Assessment (PedMIDAS) questionnaire was applied to all patients at each visit, and the total scores were computed according to the method proposed by Hershey et al. [20]. The patients were classified into the following four groups based on PedMIDAS scores: grade I, no marginal disability (0–10 days); grade II, mild disability (11–30 days); grade III, moderate disability (31–50 days); and grade IV, severe disability (≥51 days). Migraine severity was measured using the visual analog scale (VAS) [21].

The laboratory specimens of all patients were evaluated at the Biochemistry and Hormone Laboratory of the Kocaeli Derince Training and Research Hospital. The Architect c16000 clinical chemistry spectrophotometer (Abbott Diagnostics, Illinois, USA) was used for the measurements. A chemiluminescence immunoassay method with the Siemens Advia Centaur XP immunoassay system (Siemens Healthcare Diagnostics, Muenchen, Germany) was used to evaluate the serum PTH levels. High-performance liquid chromatography with the Architect i2000SR immunoassay analyzer (Abbott Diagnostics, Illinois, USA) was used to measure the serum 25-OH vitamin D levels.

Based on previous studies and reports of the Institute of Medicine, serum 25-OH vitamin D levels >20 ng/mL were considered normal, 10–20 ng/mL were insufficient, and <10 ng/mL were deficient [22].

Other normal serum values were defined as 8.8–10.8 mg/dL for Ca, as 75–400 U/L for ALP, as 2.8–6.0 mg/dL for P, and as 15–65 pg/mL for PTH, in accordance with laboratory reference rates. Patients were divided into two groups according to 25-OH vitamin D levels. Patients with 25-OH vitamin D levels <20 ng/mL were group 1 and >20 ng/mL were group 2. The PedMIDAS and VAS scores; migraine frequency; migraine duration; and 25-OH vitamin D, Ca, P, ALP, PTH levels of the patients in group 1 were compared with those of the patients in group 2. Patients in group 1 were treated with 2000 IU/day of vitamin D for 2 months and with 600–1000 IU/day of maintenance therapy for the next 6 months [23]. Those patients with hypocalcemia or high PTH levels in group 1 were also provided 30–75 mg/kg/day of calcium supplementation [24]. Patients in group 2 were monitored without vitamin D therapy. After 6 months of therapy, the obtained PedMIDAS and VAS scores of patients in each group were compared with the previously obtained scores. After vitamin D therapy, the levels of 25-OH vitamin D of group 1 compared with the earlier levels. Also, calcium, phosphorus, PTH, and ALP levels were compared with baseline levels after vitamin D therapy.

This study was approved by the Şişli Hamidiye Etfal Training and Research Hospital Ethics Committee (07.08.2018-698).

## 3. Statistical Methods

All data were analyzed using the SPSS software version 17.0 (IBM) to evaluate distributions, as appropriate. The Kolmogorov–Smirnov test was used to examine the conformity of the data to a normal distribution. The chi-square test was used to analyze categorical variables. The nonparametric Mann–Whitney *U*-test and Wilcoxon test were performed to evaluate the comparisons within quantitative variables. Spearman correlation analysis was used when the relationships between the numerical variables did not satisfy the parametric test conditions. Values were expressed as percentages or as medians. *p* < 0.05 was considered statistically significant.

## 4. Results

A total of 92 pediatric patients with migraine (67 girls (72.8%) and 25 (27.2%) boys) were included in the study. The median patient age was 12.6 (range, 6–18) years. The migraine frequency, migraine duration, and pedMIDAS scores were found to be significantly higher in patients with low vitamin D levels compared to patients with normal vitamin D levels. However, there was no difference between the groups in terms of migraine severity. Table 1 presents the patients’ characteristics.

Vitamin D insufficiency and deficiency were observed in 32 (34.7%) and 10 patients (10.8%), respectively within all patients. PTH and ALP levels were significantly higher in patients with low vitamin D. The laboratory values of the patients are given in Table 2.

The median migraine severity score was 6.9. There was no difference in the migraine severity between girls and boys. There were also no significant differences in the migraine severity, frequency, and migraine duration as well as in the PedMIDAS scores between patients with and without a family history of migraine.

We found statistically significant negative correlations between the migraine frequency, migraine duration, PedMIDAS score, and the vitamin D levels (*p* < 0.001). However, we observed no association between migraine severity and 25-OH vitamin D levels Table 3.

Migraine frequency and the PedMIDAS scores were significantly higher in the patients with vitamin D deficiency than in those with vitamin D insufficiency (*p* = 0.001; *p* < 0.001). However, we found no considerable association between the migraine severity and vitamin D level, between duration of migraine and vitamin D level, or between the patients with vitamin D insufficiency and those with vitamin D deficiency (Table 4).

The patients in group 1 reported statistically significant shorter durations of migraine after vitamin D therapy (*p* < 0.001). After 6-month of therapy, the migraine frequency and duration as well as the VAS scores were significantly decrease in the group 1 patients (*p* < 0.001; Table 5). After vitamin D treatment, PTH levels were significantly decreased in group 1 compared to baseline levels (*p* < 0.001). Calcium, phosphorus, and ALP levels were not significantly changed after treatment (*p* = 0.06, 0.09, 0.08).

## 5. Discussion

Vitamin D deficiency is widely prevalent and leads to various disorders. Low serum vitamin D levels may cause chronic pain and headache disorders, specifically tension-type headache and migraine [14,25,26].

Through our survey, we observed notable decreases in the PedMIDAS score as well as the migraine duration, frequency, and severity in the patients who received vitamin D therapy.

Vitamin D deficiency was defined based on various references. In most studies, vitamin D plasma levels <10 ng/mL are considered a deficiency; however, defining the threshold for the level of vitamin D insufficiency remains a controversial issue [22,24,27]. Also, most studies have reported that complications related to low levels of vitamin D, such as hypoparathyroidism, bone fractures, and multiple sclerosis, are more frequent at levels <20 ng/mL [3,28]. According to the Institute of Medicine, 20 ng/mL (50 nmol/L) is the adequate level for at least 97.5% of the total population and clinicians should consider this level when managing patients [22,28].

Many authors have reported that a 30 ng/mL level of 25-OH vitamin D plasma is optimal [3]. Mithal et al. reported that there is an ongoing global debate on the definition of deficient or insufficient vitamin D levels; furthermore, there is currently no standard definition for the optimal vitamin D status. A vitamin D level of 30 ng/mL may be inappropriate as a normal cutoff; in that case, vitamin D inadequacy has been overestimated [22,27]. For our study, we defined 25-OH vitamin D levels >20 ng/mL as normal, levels between 10–20 ng/mL as insufficient, and levels <10 ng/mL as deficient.

More extreme vitamin D deficiencies and more severe pain in female patients have been reported in previous studies [12,29]. We found that, for patients in both groups, most patients with low vitamin D levels were female; however, no statistically significant difference was noted (0.506).

Children aged 9–18 years experience growth spurts characterized by a remarkable increase in their requirements for phosphorus and calcium to escalate skeletal mineralization. For metabolism, more vitamin D is needed during puberty [30]. Knutsen et al. established the relationship between age and the severity of vitamin D deficiency [30]. No significant relationship was observed between the 25-OH vitamin D levels and age (*p* = 0.077).

Vitamin D is crucial in mineral homeostasis and comprises an anti-inflammatory hormone that regulates endothelial functions, immune responses, and cell proliferation. [31,32] Furthermore, vitamin D plays a negative role in the proliferation of mast cells, regulates nitric oxide production, and inhibits inducible nitric oxide synthase (iNOS) expression (Nitric oxide is a vasoactive substance and is used by blood vessels.) [33]. Vitamin D deficiency causes inflammation because of the dysregulation of nitric oxide production, which adversely affects immune responses, cell migration, and apoptosis [14,33]. Moreover, in chronic pain and migraine attacks, the levels of substance P, the calcitonin gene-related peptide (GCRP), and neuroexcitator mediator increase [34,35]. Vitamin D therapy causes a decline in the levels of these factors as well oxidative stress.

Vitamin D deficiency can cause secondary hyperparathyroidism. Some studies have reported that the levels of PTH, which is associated with endothelial dysfunction, are higher in the systemic circulation of patients with heart failure [36]. Also, PTH upregulates the activity of the endothelial nitric oxide synthase system via protein kinase pathways [37].

Vitamin D may lead to the inhibition or stimulation of the expression of several calcium channels, and this may cause neuronal hyperexcitability [38]. Vitamin D may stimulate serotonin synthesis via tyrosine hydroxylase; hence, vitamin D deficiency may lead to the depression of serotonin synthesis, which is usually observed in tension-type headache and migraine [12].

Vitamin D inhibits matrix metalloproteinase 9 (MMP-9) in humans. MMP-9 is a proteolytic enzyme; these enzymes improve endothelial dysfunction [39]. Few activities of intracellular signal pathways are prevented by vitamin D. Extravascular inflammatory pain, such as that produced by migraine, stems from vitamin D deficiency [8]. Furthermore, low vitamin D levels result in hypomagnesemia, which plays a role in chronic pain [14].

Yang et al. highlighted a potential correlation between vitamin D deficiency and headaches [40]. In 4061 adult patients, Kjaergaard et al. reported a link between vitamin D and tension-type headache but could not propose a correlation between vitamin D and migraine [41].

Thys-Jacobs et al. stated that supplemental calcium and vitamin D administration produced a sudden reduction in the migraine duration and frequency [13]. The results could be attributed to both calcium and vitamin D levels; the two could have also enhanced the absorption of magnesium, which in turn may have led to a decrease in migraine headaches [13,15].

O’Brien et al. also documented a high prevalence of vitamin D deficiency among 300 pediatric patients with migraines [16]. Moreover, they discovered that the prevalence of vitamin D deficiency was considerably higher among people with recurrent headaches than among those without such headaches.

Prakash et al. reported the positive effects of calcium and vitamin D supplements on tension-type headache [14]. Prakash et al. also reported high levels of PTH in patients with migraines, which could be due to secondary hyperparathyroidism that was treated by optimal levels of vitamin D supplements [26].

Knutsen et al. reported that vitamin D levels have a much stronger relationship with headache than with either musculoskeletal pain or fatigue [29]. Zandifer et al. examined 105 patients aged 15–65 years with migraines and set up a control group of 110 patients [42]. They basically compared the 25-OH vitamin D levels; however, no statistically significant association was observed between migraines and vitamin D levels.

After studying 147 patients aged 5–16 years with headaches (either tension-type or migraine) and 69 controls, Dönmez et al. reported that, in conditions of low solar exposure, the relationship between headache and vitamin D deficiency was of limited significance [18].

Cayır et al. studied 53 children aged 8–16 years and reported that vitamin D therapy with amitriptyline reduces the recurrence of migraine attacks in pediatric patients with migraine [17].

To the best of our knowledge, this is the first case–control evaluation of vitamin D and the response to vitamin D therapy in pediatric patients suffering from migraine. We detected significant negative relationships between the migraine frequency and serum vitamin D levels, as well as between migraine duration and PedMIDAS scores (r = −0.591, *p* < 0.001; r = −0.437, *p* < 0.001; and r = −0.644 *p* < 0.001, respectively). However, no association was observed (*p* = 0.128) between the migraine severity and vitamin D levels. After treatment with vitamin D, migraine severity (VAS scores), migraine duration, migraine frequency, and PedMIDAS scores were significantly decreased in the treated patients.

Limitation of this study may be the fact that no data about the patients’ lifestyle, geographical location, and socioeconomic status were available and the fact that vitamin D levels were not compared between patients diagnosed with migraine and a healthy control group.

## 6. Conclusions

We found a significant relationship between pediatric migraine and vitamin D levels. We also demonstrated that vitamin D therapy (supplemented with calcium) produced favorable effects on pediatric patients. Thus, vitamin D supplementation can help improve the quality of life of pediatric patients suffering from migraine. Randomized clinical trials may help further establish this conclusion. Survey studies executed with more subjects, as well as more control groups for longer durations that include pre-evaluations may provide a better understanding of this topic.

## Figures and Tables

**Table 1:** Characteristics of group 1 and group 2.

Characteristic	Group 1 (n = 42)	Group 2 (n = 50)	*p*
Age, median (range), years	14 (7–18)	12.2 (6–16.6)	0.077 *
Gender, n (%)			
Girls	32 (76.2)	35 (70.0)	0.506 **
Boys	10 (23.8)	15 (30.0)	
Family history, n (%)	27 (64.3)	35 (70.0)	0.560 **
Migraine severity, median (range), VAS score	6.5 (3–10)	6.0 (3–10)	0.106 *
Migraine frequency, median (range), number of attacks/month	11.8 (1–30)	5.7 (1–15)	0.004 *
Migraine duration, median (range), hours/day	10.8 (1–72)	3.3 (1–8)	0.008 *
PedMIDAS, median (range), score	21.4 (1–83)	5.8 (1–35)	0.001 *
PedMIDAS score by grade, n (%)			
Grade I	21 (50.0)	28 (56.0)	
Grade II	11 (26.2)	21 (42.0)	0.003 **
Grade III	4 (9.5)	1 (2.0)	
Grade IV	6 (14.3)	0 (0.0)	

PedMIDAS: Pediatric Migraine Disability Assessment. * Mann–Whitney *U*-test was used. ** Chi-square test was used.

**Table 2:** Baseline laboratory parameters of patients in group 1 and group 2.

Parameter	Group 1	Group 2	*p*
25OH vit D, ng/mL	9.4 (4.2–20)	32.6 (21–45)	<0.001 *
Ca, mg/dL	9.5 (8.8–10.2)	9.4 (8.8–10.1)	0.501 *
P, mg/dL	4.1 (2.9–5.3)	4.2 (3.2–5.3)	0.418 *
ALP, U/L	153.6 (107–256)	115.7 (45–190)	0.044 *
PTH, pg/mL	74.3 (33.8–139)	42.7 (25–67)	<0.001 *

Values are median (range). ALP: Alkaline phosphatase, Ca: Calcium, P: Phosphorus, PTH: Parathormone. * Mann–Whitney *U*-test was used.

**Table 3:** Correlations between the vitamin D level of the patients and the migraine severity, migraine duration, migraine frequency, and PedMIDAS score.

	25-OH vitamin D	
	*r* *	*p*
Migraine severity (VAS score)	−0.195	0.128
Migraine duration (hour/day)	−0.437	**<0.001**
Migraine frequency (attack number/month)	−0.591	**<0.001**
PedMIDAS score	−0.644	**<0.001**

PedMIDAS: Pediatric Migraine Disability Assessment. * Spearman correlation analysis was used.

**Table 4:** Relationship between Vitamin D levels and migraine severity, duration, frequency, and PedMIDAS score.

	25-OH vitamin D, ng/mL		*p* *
	<10	10–20	
Migraine severity, VAS score	6.6 (3–10)	6.9 (3–10)	0.564
Migraine duration, hours/day	11.07 (1–72)	9.2 (1–72)	0.596
Migraine frequency, number of attacks/month	14.1 (1–30)	7.0 (1–20)	**0.001**
PedMIDAS, score	26.0 (1–83)	7.4 (1–45)	**<0.001**

Values are median (range). PedMIDAS: Pediatric Migraine Disability Assessment. * Mann–Whitney *U*-test was used.

**Table 5:** Migraine frequency, duration, VAS score, and PedMIDAS score after vitamin D treatment in group 1 patients.

Variable	Before Treatment	After Treatment	*p* *
25-OH vit D level, ng/mL	9.4 (4.2–20)	34.6 (16.3–45)	**<0.001**
Migraine duration, hours/day	10.8 (1–72)	2.5 (0–12)	**<0.001**
Migraine frequency, number of attacks/month	11.8 (1–30)	4.5 (0–15)	**<0.001**
Migraine severity, VAS score	6.7 (3–10)	3.6 (3–6)	**<0.001**
PedMIDAS, score	21.4 (1–83)	5.6 (0–30)	**<0.001**

Values are median (range). PedMIDAS: Pediatric Migraine Disability Assessment, VAS: Visual Analog Scale. * Wilcoxon test was used.
